# Prolapsus rectal étranglé de l’adulte jeune: à propos d’un cas et revue de la littérature

**DOI:** 10.11604/pamj.2016.25.60.10721

**Published:** 2016-10-03

**Authors:** Rached Bayar, Achref Djebbi, Zeineb Mzoughi, Ghofrane Talbi, Lassaad Gharbi, Nafaa Arfa, Hafedh Mestiri, Mohamed Taher Khalfallah

**Affiliations:** 1 Université de Tunis El Manar, Faculté de Médecine de Tunis, 1007, Tunis, Tunisie; 2 Service de Chirurgie Viscérale CHU Mongi Slim, Sidi Daoued La Marsa, Tunisie

**Keywords:** Prolapsus rectal, adulte jeune, urgence, opérations périnéales, Rectal prolapse, young adult, urgency, perineal operations

## Abstract

Le prolapsus rectal est un trouble de la statique du rectum, qui réalise une invagination de la paroi rectale aboutissant à son extériorisation à travers l’anus. Il touche généralement l’enfant et le sujet âgé. Sa survenue chez l’adulte jeune est rare. L’étranglement du rectum prolabé est également une complication rare. Nous rapportons l’observation d’un jeune de 30 ans, opéré en urgence pour un prolapsus rectal étranglé. Il a eu une recto-sigmoïdectomie périnéale (intervention d’Altemeier) en urgence avec des suites simples.

## Abstract (en)

Rectal prolapse is a rectal static disorder which involves rectal wall intussusception inducing its externalization through the anus. It usually affects children and the elderly. Its occurrence in young adults is rare. Strangulated rectal prolapse is also a rare complication. We report the case of a 30-year old patient who underwent emergency surgery for strangulated rectal prolapse. Emergency perineal rectosigmoidectomy (Altemeier repair) was performed with simple outcome.

## Introduction

Le prolapsus rectal (PR) est la protrusion du rectum à travers l’anus. Il s’agit d’une affection qui touche essentiellement les enfants âgés de 1 à 3 ans et les sujets âgés. Sa survenue chez l’adulte âgé de moins de 30 ans est rare, comme en témoigne le manque de publications sur le sujet dans cette population. L’étranglement du PR est une complication rare qui survient dans 2 à 4% des cas [1].

## Patient et observation

**Figure 1:**
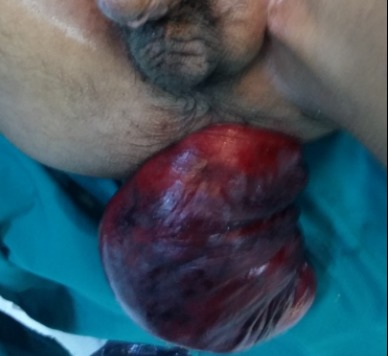
Prolapsus rectal complet avec des plages de nécrose

**Figure 2:**
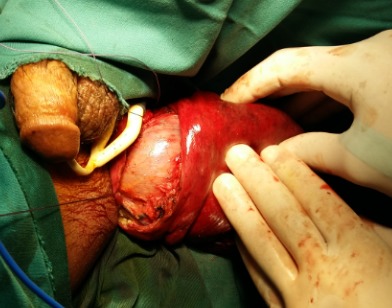
Section muqueuse juste au-dessus de la ligne pectinée. Muqueuse rectale, séreuse colique

**Figure 3:**
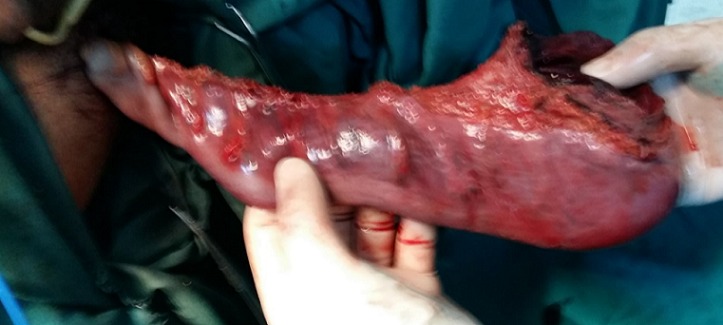
Colon abaissé en trans anal

**Figure 4:**
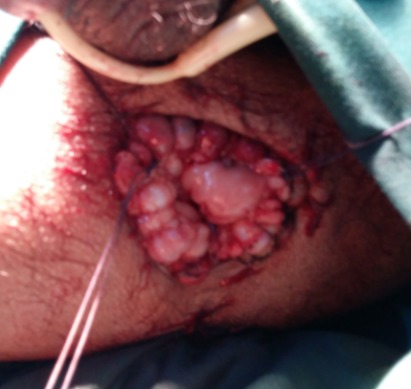
Anastomose colo anale

Nous rapportons le cas d’un patient âgé de 30 ans, atteint d’une infirmité motrice cérébrale (IMC), qui consulte aux urgences pour un prolapsus rectal extériorisé et non réductible depuis 6 jours. On note dans ses antécédents plusieurs épisodes d’extériorisations réduites par des manœuvres digitales. A l’examen, le patient était fébrile à 38°C avec un état hémodynamique stable. Il avait un prolapsus rectal irréductible nécrosé (Figure 1). Le patient était opéré en urgence. Il avait eu une recto-sigmoïdectomie avec anastomose colo-anale par voie périnéale selon la technique d’Altemeier: le rectum était sectionné juste au-dessus de la ligne pectinée (Figure 2), le colon abaissé en transanal était sectionné en regard de la ligne pectinée (Figure 3). L’anastomose colo-anale était réalisée manuellement par des points séparés (Figure 4). Une lame ondulée, trans-anastomotique, était laissée en place. L’anastomose n’a pas été protégée en raison de l’IMC. Les suites opératoires étaient simples. Le patient va bien avec un recul de 6 mois.

## Discussion

Le prolapsus rectal est une pathologie fréquente chez les enfants et les personnes âgées. Son incidence dans la population d’adultes jeunes de moins de 30 ans est exceptionnelle. Les causes du PR restent peu connues. Dans la littérature, il n’existe pas de cause évidente qui explique à elle seule la survenue du prolapsus rectal [1]. Les facteurs associés au PR sont un âge avancé, une multiparité chez les femmes, une dysfonction du plancher pelvien ou une lésion du périnée. Les troubles de défécation et la dyschésie avec poussées prolongées associés à la constipation sont des causes fréquentes de PR chez l’enfant [1]. Dans le cas de notre patient, une constipation chronique non explorée tait notée. Elle serait due à l’IMC et serait à l’origine du PR. En effet, selon Campanosie [1], la constipation était présente chez 67% des enfants avec IMC.

Le prolapsus peut être spontané ou provoqué par la station debout ou par la toux. D’autres symptômes, parfois associés à la constipation, peuvent révéler le PR. Ce sont l’évacuation incomplète, le saignement rectal, la douleur rectale, l’incontinence, le besoin impératif d’aller à la selle et les ténesmes. L’étranglement du prolapsus rectal constitue une complication rare qui survient dans 2 à 4% des cas [2]. La nécrose du segment incarcéré reste une complication exceptionnelle. Lorsque le rectum incarcéré ne peut être réduit, quelques techniques peuvent aider à débloquer la situation comme la sédation et l’application de sel et de sucrose permettant ainsi de réduire l’œdème et de réduire le prolapsus [3]. En cas d’échec de ces procédés ou en cas de nécrose, l’intervention chirurgicale devient urgente [3].

Plusieurs interventions chirurgicales sont décrites pour le traitement du PR par voie abdominale et par voie périnéale. Le but de ce traitement est de restaurer une position anatomique normale du tube digestif et d’améliorer les signes fonctionnels. Le choix du traitement initial dépend de la présentation clinique et de l’expérience du chirurgien [4]. En dehors des situations d’urgence, l’approche abdominale (rectopexies, résections coliques et colorectales ou l’association des deux) semble donner moins de récidives [2, 5] mais elle est à éviter chez le sujet jeune vu le risque ultérieur d’infertilité. En situation d’urgence, seule la résection recto-sigmoïdienne par voie périnéale ou procédé d’Altemeier peut être proposée avec ou sans colostomie [2]. L’intervention de Delorme est difficile dans cette situation à cause de l’œdème et elle est contre indiquée en cas de nécrose [6]. La morbidité post opératoire immédiate pour l’intervention d’Altemeier, réalisée en urgence, est quasiment nulle avec un très faible risque de lâchage anastomotique [7]. A long terme, le risque de récidive reste cependant plus élevé que celui des techniques d’abord abdominal [7].

## Conclusion

Le prolapsus rectal étranglé est une complication rare. La nécrose du segment extériorisé est exceptionnelle mais grave pouvant engager le pronostic vital. L’intervention d’Altemeier est l’intervention de choix dans cette situation. Le taux de lâchage est quasi nul mais le risque de récidive à long terme est important.
